# Chronic multiscale resolution of mouse brain networks using combined mesoscale cortical imaging and subcortical fiber photometry

**DOI:** 10.1117/1.NPh.10.1.015001

**Published:** 2023-01-21

**Authors:** Daniel Ramandi, Nicholas J. Michelson, Lynn A. Raymond, Timothy H. Murphy

**Affiliations:** University of British Columbia, Djavad Mowafaghian Centre for Brain Health, Department of Psychiatry, Vancouver, British Columbia, Canada

**Keywords:** calcium imaging, corticostriatal pathway, mesoscale imaging, fiber photometry

## Abstract

**Significance:**

Genetically encoded optical probes to image calcium levels in neurons *in vivo* are used widely as a real-time measure of neuronal activity in the brain. Mesoscale calcium imaging through a cranial window provides a method of studying the interaction of circuit activity between cortical areas but lacks access to subcortical regions.

**Aim:**

We have developed an optical and surgical preparation that preserves wide-field imaging of the cortical surface while also permitting access to specific subcortical networks.

**Approach:**

This was achieved using an optical fiber implanted in the striatum, along with a bilateral widefield cranial window, enabling simultaneous mesoscale cortical imaging and subcortical fiber photometry recording of calcium signals in a transgenic animal expressing GCaMP. Subcortical signals were collected from the dorsal regions of the striatum. We combined this approach with multiple sensory-motor tasks, including specific auditory and visual stimulation, and video monitoring of animal movements and pupillometry during head-fixed behaviors.

**Results:**

We found high correlations between cortical and striatal activity in response to sensory stimulation or movement. Furthermore, spontaneous activity recordings revealed that specific motifs of cortical activity are correlated with presynaptic activity recorded in the striatum, enabling us to select for corticostriatal activity motifs.

**Conclusion:**

We believe that this method can be utilized to reveal not only global patterns but also cell-specific connectivity that provides insight into corticobasal ganglia circuit organization.

## 1 Introduction

The multiscale characteristics of cortical activity have been defined through macro, meso, and micro scales of recording.^1^ The local and global activity recorded through wide-field optical imaging of the cortex in animals expressing genetically encoded ${\mathrm{Ca}}^{2+}$ indicators (GCaMPs) enables the study of the behaviorally relevant computations in the cortex across scales that reveal interactions between cortical areas.^2^ Although this mesoscale approach can measure neural activity over a broad area of the cortex, it cannot capture the activity of subcortical structures and connections. Subcortical imaging can be achieved through surgical aspiration of tissue, penetrating gradient-index (GRIN) lenses, or 3-photon microscopy, all of which involve sophisticated recording devices and may lead to damage to a cortical region in the process of implementation. In fact, cortical tissue aspiration (which is mainly used for GRIN lens implantation) can cause damage to subcortical regions as well.^3^

Recently, there have been advancements in experimental techniques that enable simultaneous recording of neural activity in the cortex and subcortical regions using cortex-wide epifluorescence ${\mathrm{Ca}}^{2+}$ imaging, coupled with whole-brain functional magnetic resonance imaging (fMRI),^4^ or high-density electrode arrays (neuropixels silicon probes).^5^^–^^7^ The former requires a relatively strong MRI machine and still lacks resolution of specific cell populations through genetically encoded indicators, and utilizing the latter for chronic preparations is challenging.

A simple yet powerful technique to record point source fluorescence activity from deep brain regions is fiber photometry.^8^ Using this method, multiple fluorescent indicators are excited and the signals are collected at the tip of one or multiple implanted optical fibers.^9^ Although it lacks cellular resolution, its versatility and the possibility to implant optical fibers chronically allow researchers to use this method in combination with any cell-type specific fluorescent indicators for long-term recordings. The implanted fibers can also be used for optogenetic perturbations, allowing scalable brain-wide manipulations as well as recordings.

Here, we describe a multiscale approach to study the interactions between the mouse cortex and striatum. We use a chronic cortical window preparation over an intact skull^10^ and a diagonally implanted fiber in the dorsal striatum of a transgenic mouse model expressing GCaMP6s in the cortical projection neurons (thy1.GCaMP) to measure calcium signals in the cortex (using wide-field mesoscale cortical imaging) and striatal axonal terminals (using fiber photometry).^11^

The objectives of this study are threefold: (1) develop a robust and safe surgical preparation for multiscale recording of the corticostriatal activity, (2) create reference correlation maps that represent the best estimate of the mouse corticostriatal functional connectivity, and (3) identify global patterns/motifs that provide insight into corticostriatal circuit organization.

## 2 Methods

### 2.1 Animals

All procedures were approved by The University of British Columbia Animal Care Committee and conformed to the Canadian Council of Animal Care and Use guidelines (protocol A22-0054). The experiment was performed on transgenic female mice (3- to 4-months old) expressing the calcium indicator GCaMP6s driven by the Thy1 promoter (Thy1-GCaMP6s; Jackson mouse #024275, GP4.3, C57BL/6-Tg(Thy1-GCaMP6s)GP4.3Dkim/J16). The expression of GCaMP6s was determined by polymerase chain reaction genotyping on each animal and confirmed by histology after the experimental endpoint.

### 2.2 Surgical Procedures

**Fig. 1:**
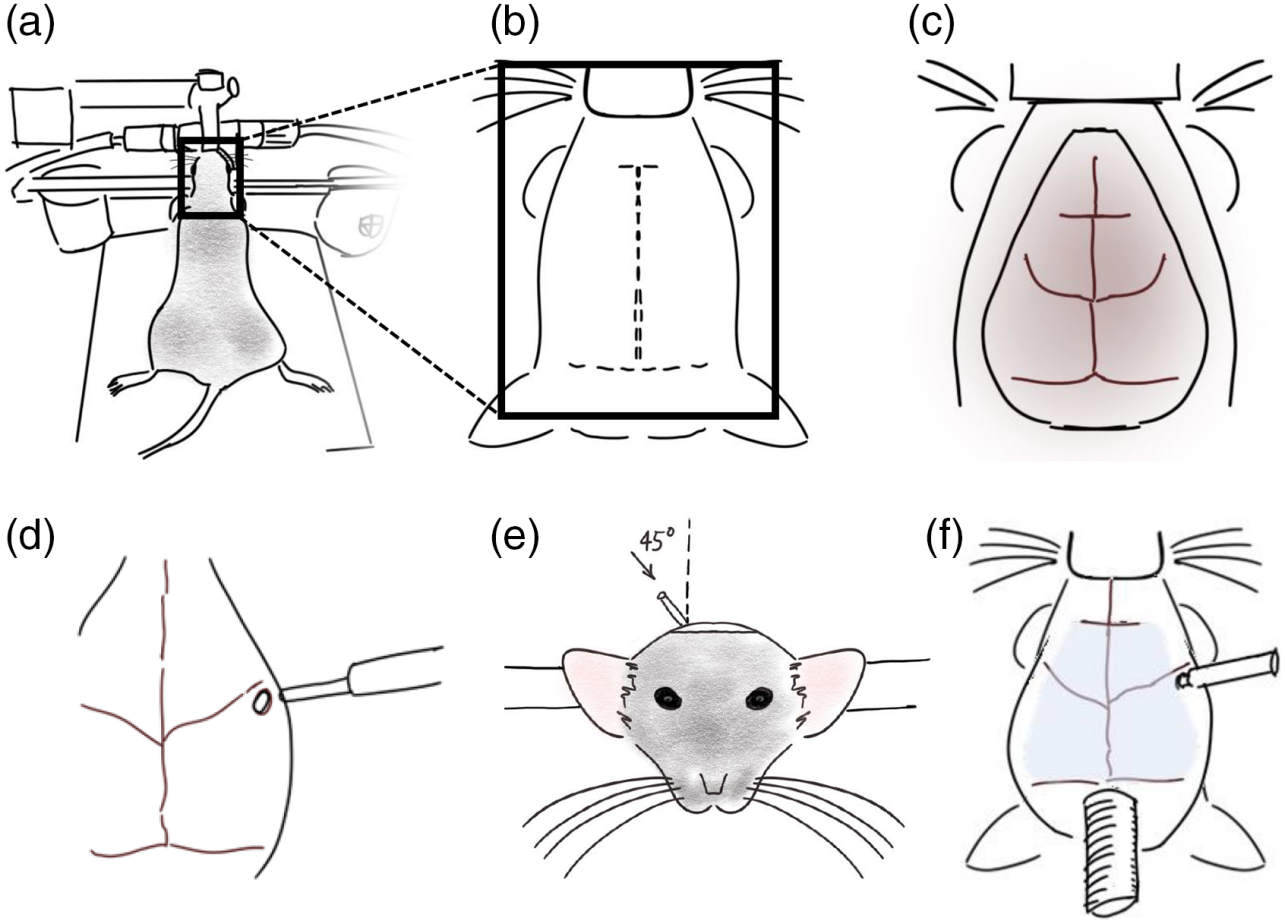
Surgical implantation of optical fiber and cranial window. Steps described in the methods section. The animals were placed in a stereotactic frame (a), and the fur and skin over the skull were removed (b) to expose the surface of the skull (c). The optical fiber implant was inserted through a drilled hole (d) at a 45-degree angle (e). Finally, the glass cranial window and a head screw (f) were fixed in place using clear dental cement.

Before starting the surgery, a No. 1 circular coverglass (Marienfeld, Lauda-Konigshofen, Germany; Cat#:0111520) was cut with a diamond pen to the size of the final cranial window (∼9-mm diameter). Large transcranial glass windows were installed on the intact skull of mice, as described previously.^10^ Briefly, for this procedure, mice were anesthetized with 2% isoflurane and maintained with 1.5% isoflurane in air, and placed in a stereotactic frame [Fig. 1(a)]. Using a rectal probe and a feedback-regulated heating pad, the body temperature was maintained at 37°C. Local analgesic was applied through an injection of lidocaine (0.1 ml, 0.2%) under the scalp. The mice were also administered subcutaneously with a saline solution containing buprenorphine (2  mg/ml), atropine (3  μg/ml), and glucose (20 mM). The fur on the top of the head was removed using surgical scissors, and a triple scrub of 0.1% betadine in water followed by 70% ethanol was applied. The skin on the top of the head was cut [Fig. 1(b)] and removed, and fascia and connective tissues on the surface of the skull were removed so that the skull surface was completely clear of debris and dry [Fig. 1(c)]. Before placing the glass cover on the skull, a silica/polymer mono-fiber optic cannula was inserted into the right dorsal striatum. The cannula consisted of a 3-mm flat-tip fiber with a core diameter of 400  μm (0.48 NA), attached to a 1.25-mm metal ferrule (Doric Optics; Ordering Code: MFC_400/430-0.48_3.0 mm_MF1.25_FLT). Using a micromanipulator (Sutter; Model 285A) mounted on the stereotaxic apparatus, the location of the fiber entering the brain was marked on the skull (right hemisphere) and drilled (∼1-mm diameter) with a dental drill [Fig. 1(d)]. The location is in reference to the mouse brain atlas (AP +0.1, ML +3  mm). The fiber was inserted through the hole, at a 45-deg angle 2750 to 2800  μm deep [Fig. 1(e)], and the ferrule was fixed in place with Krazy glue. To head-fix the animals for cortical imaging, a stainless 4 to 30 stainless steel set-screw was also glued to the cerebellar plate, directly posterior to lambda [Fig. 1(f)]. Finally, clear dental cement, prepared by mixing 1 scoop of Metabond powder, 6 drops of C&B Metabond Quick Base, and one drop of C&B Universal catalyst (Parkell, Edgewood, New York) was applied directly on the skull, and a precut cover glass was placed on top of the mixture before it solidified. The dental adhesive was added around the fiber and screw to hold everything firmly in place. The animals’ weight and health were monitored daily for one week after surgery and none of the animals showed any detectable signs of damage to the jaw muscles or eye. The weight loss following the surgical procedure did not exceed 10% of the presurgical weight and returned to baseline within 5 to 7 days post-surgery.

### 2.3 Fiber Photometry Recording

*In vivo* striatal photometry data were acquired by a custom-built fiber photometry rig. Illumination was provided by 405- and 465-nm fiber collimated LEDs (Doric Lenses; Models CLED_405/465), controlled and modulated through an LED driver (Doric Lenses; Model LEDD_4). The LEDs were connected to a 6-port fluorescence minicube (Doric Lenses; FMC6). A fiber optic patch cord was used to connect to the fiber implant and deliver light to excite and record the GCaMP6 signal. The excitation LED power measured at the end of the optical fiber patch cord was 150 to 200  μW. The calcium-insensitive fluorescence signal (GCaMP isosbestic point; excitation: 405-nm modulated at 210 Hz) and calcium-dependent fluorescence (GCaMP excitation: 465-nm modulated at 320 Hz) were collected and measured using the mini-cube output connected to a 2151 Femtowatt Photoreceiver (Newport). The signal was digitized at ∼1  kHz with an RZ5P signal processor [Tucker- Davis Technologies (TDT)], controlled by Synapse software (TDT).

Stimulus and task event timestamps were recorded through Transistor–Transistor Logic (TTL) signals generated by the stimulator and integrated with the fiber photometry signals through the RZ5P signal processor digital inputs. A custom MATLAB script (MATLAB R2021a, Mathworks) was written to extract, filter, and analyze the data. All recordings showed calcium peaks (recorded through the optical fiber) correlated to behavioral events confirming that recordings without detectable peaks were not due to technical issues.

To normalize the photometry data, we employed two methods: (method 1) the calcium-insensitive isosbestic channel (405 nm) was fitted to the calcium sensitive 465-nm channel, and treated as baseline fluorescence F0. F0 was then subtracted from the raw trace (ΔF) and then divided from the resulting difference, giving the ΔF/F0; or (method 2) a 10-s moving average of the raw trace was calculated and subtracted from the signal, giving the ΔF/F. Method 1 is mainly used when animals are freely moving, to remove any movement artifacts. As the animals were head-fixed during the experiments, minimal movement artifacts were detected (as defined by an isosbestic point fiber signal) and both methods yielded similar results. The moving average method was preferred, as the same method was used to calculate the ΔF/F of the mesoscale cortical GCaMP recordings and was used to minimize the impact of potential low-frequency global activity.

### 2.4 Mesoscale Cortical Recording

About 10 to 14 days following surgery, all mice were habituated to being head-fixed in the imaging apparatus. The mice were rewarded with peanut butter following all imaging sessions. To minimize anxiety induced by 20 min of being head-fixed for experimental sessions, the habituation sessions (3 sessions for each animal) were 2 to 5 min each and incrementally longer for consecutive sessions. Awake resting-state spontaneous cortical activity was recorded in a long 20-min trial. The visual and auditory evoked activity were recorded during 5 to 10 min trials. A behavioral camera (Raspberry Pi camera, NoIR with adjustable focus) with an infrared (IR) bandpass filter, and an IR light were placed inside the imaging chamber to monitor active behaviors and pupil diameter. A python script was written that generated the TTLs for timelocking the signals and recording the behavior video. The behavior video was recorded with a resolution of 500×500  pixels at 10 frames per second.

To evoke visual responses, a blue (470-nm LUXEON Rebel) light-emitting diode (LED) was placed ∼2  cm from the eye (∼45-deg azimuth, ∼0-deg elevation). The LED was connected to an isolated pulse stimulator (A-M Systems Model 2100). The LED was illuminated with a 3.3 V pulse for a duration of 10 ms with an interpulse interval of 10 s. Sensory responses to the auditory stimuli were evoked using a monotone 12-kHz beep, produced using a 10-ms pulse width modulated (PWM) output from the Raspberry Pi to a piezo buzzer (Adafruit PID #1739). The PWM command was generated by the same Raspberry Pi that collected the behavior video. About 40 consecutive tones with an inter-stimulus interval (ISI) of 10 s were delivered during the imaging session. With each PWM command for auditory stimulation or LED flash for visual stimulation, a TTL high signal (3.3 V) was sent from the Raspberry Pi board to the photometry signal processor to record the epoch timestamps.

**Fig. 2:**
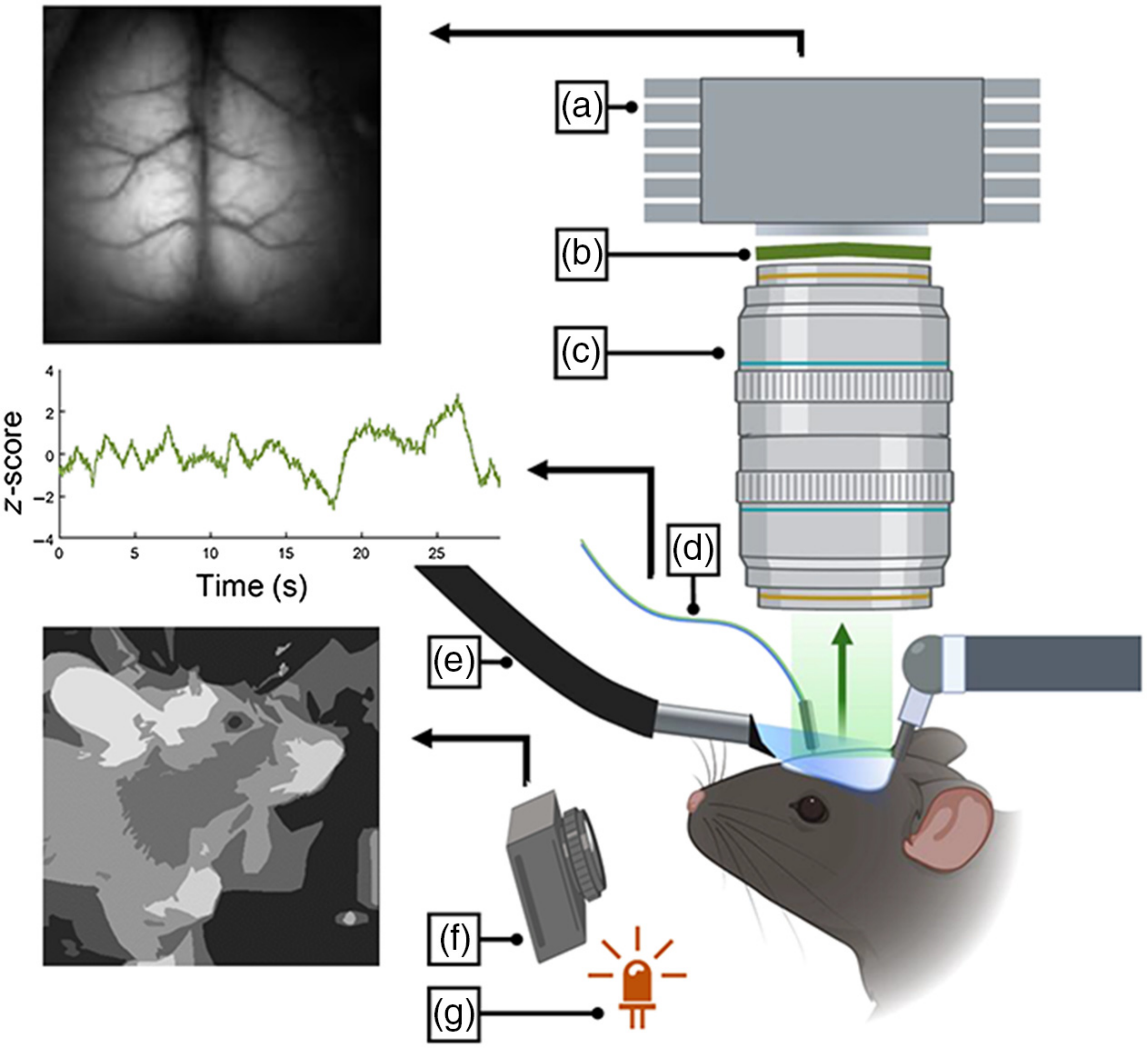
Diagram of the imaging apparatus. (a) Monochromatic Pantera CCD Camera (Dalsa); (b) emission filter (green: 525/36  nm); (c) front-to-front Nikon lenses (f=1.4,35  mm; f=2); (d) optical patch cord (from brain implanted fiber, to filter cube and photodetectors); (e) light guide (Ex LED, Blue: 480/30  nm); (f) NoIR Pi Camera with IR passing filter; and (g) IR LED (850 nm).

Cortical fluorescence activity was recorded using a Pantera 1M60 CCD camera [Dalsa, Fig. 2(a)], equipped with two front-to-front lenses [50 mm, f=1.4∶35  mm, f=2; Nikon Nikkor, Fig. 2(c)] and a bandpass emission filter [525/36  nm, Chroma; Fig. 2(b)]. The 12-bit images were captured at a frame rate of 40 Hz (exposure time of 25 ms) with 8×8 on-chip spatial binning using EPIX XCAP imaging software. The cortex was illuminated with a blue LED (470 nm LUXEON Rebel) with a bandpass filter (Chroma AT480/30×) through a fiber optic light guide (Thorlabs LLG03-4H) attached to an eye shield to direct the light to the cortical window surface and eliminate light from directly entering the animal’s eyes [Fig. 2(e)]. The light guide [depicted in Fig 2(e)] provides the excitation light for the cortical imaging. Our widefield imaging system is relatively simplistic and does not employ a dichroic mirror and requires fiber delivery. The cortical excitation light intensity (at the surface of the window) was 15 to $20\;\mu W/{\mathrm{mm}}^{2}$.

To minimize post-experiment image registration later, bregma was placed in the middle of the field of view. Atlas-to-brain registration and ROI mapping were done offline using the previously described MesoNet algorithm.^12^

### 2.5 Histology

**Fig. 3:**
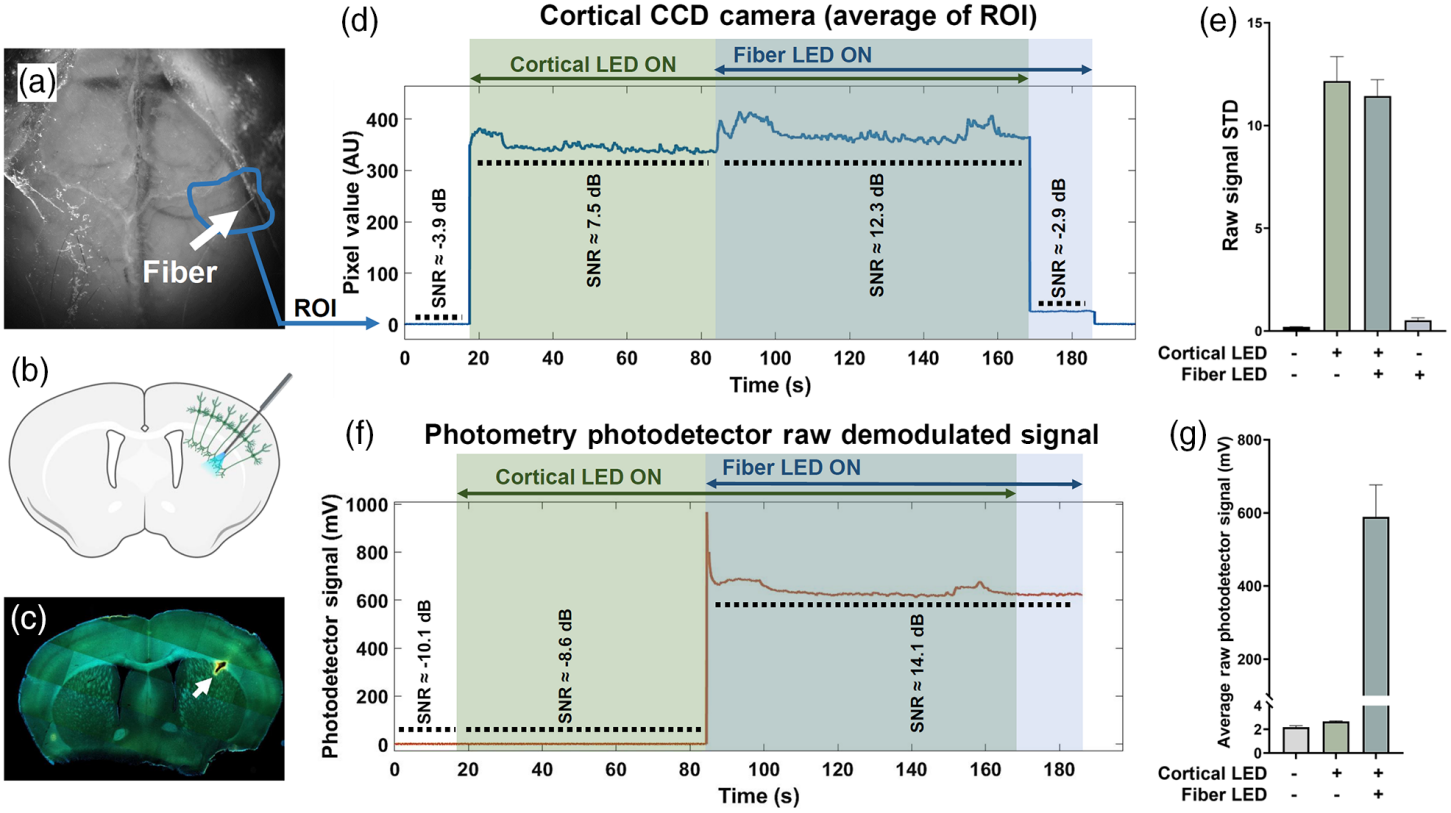
Control experiments. (a) Cortical image (white-light image without bandpass emission filter) showing fiber insertion site and region-of-interest (ROI) averaged for control experiments. (b) Diagram of a coronal brain slice of a Thy1-GCaMP mouse with cortical projection neurons expressing GCaMP6s. The cortical soma and dendritic calcium activity is captured by the widefield imaging, whereas the calcium activity of the axon terminals in the striatum is recorded through fiber photometry. (c) Coronal brain sections, showing fiber insertion site for Thy-1 GCAMP6 mice. As expected, GCaMP6s is expressed in cortical projection neurons, axon bundles and terminals in the striatum (white arrow). (d) A representative trace of the control experiments, showing raw CCD camera pixel values (average of the ROI) during Cortical and/or Fiber LED turned on, with the average SNR for the selected region. (e) The standard deviation of the cortical signal for the duration of cortical and/or fiber LEDs being on or off. (f) A representative trace of the control experiments, showing raw fiber photometry photodetector signal during cortical and/or fiber LEDs turned on, with the average SNR. (g) The average of the raw photometry signal for the duration of cortical and/or fiber LEDs being on or off.

Expression of GCaMP and location of the implanted fiber cannula were verified with postmortem histology [Figs. 3(b) and 3(c)]. Animals were euthanized (injection of pentobarbital sodium; 240  mg/kg) and perfused with phosphate-buffered saline (PBS) followed by ice-cold 4% Paraformaldehyde (PFA, dissolved in PBS). The brain was transferred to a 4% PFA solution and was left overnight. Coronal brain sections (100-μm thickness) were made using a vibratome (Leica VT1000S) and mounted onto coverslips with DAPI-Mounting Media (Fluoroshield with DAPI; Sigma). Microscopic tiled images were acquired using a Zeiss Observer Z1 fluorescence microscope (5× /0.15 NA) with GFP and DAPI filter sets. The brightness and contrast of the images were adjusted using ImageJ (Fiji).

### 2.6 Data Analysis

All the data analyses were done using MATLAB. The cortical images were imported as X/Y arrays across time [Fig. 6(a)], and ΔF/F was calculated using a 10 s moving average as F0. The signal was then spatially smoothed using a moving disc filter (diameter = 3 pixels), then temporally filtered using a second-order 0.5-dB ripple Chebyshev filter (0.1 to 12 Hz). For visually evoked recordings, the stimulation artifact was removed using linear interpolation on the raw data. The ΔF/F of the photometry signal was calculated and filtered the same way to reduce filtering-induced lags in the signals. To extract motifs of spontaneous activity, a sequential non-negative matrix factorization (seq-NMF; Figs. 6(f)–6(h) algorithm was used.^13^ Seq-NMF is an approach for decomposing changes in cortical activity into a series of major spatiotemporal activity motifs. We have used this method to validate the correlation maps observed between cortical and (presynaptic) striatal activity.

For all the correlation analyses, the z-score of ΔF/F was shown. All the signals were decimated and/or resampled (linear interpolation) to the cortical images (40 Hz), with a built-in anti-aliasing filter (low-pass Chebyshev Type I IIR filter of order 8).

The signal-to-noise ratio (SNR; in dB) was calculated using the MATLAB “snr” function (ratio of the summed squared magnitude of the signal to that of the noise).

The behavioral videos recorded through the Raspberry Pi camera were analyzed using FaceMap,^14^ to extract pupil diameter, and motion in multiple ROIs, including forelimbs. The output motion signal from FaceMap was low-pass filtered using a 1s moving average, and the z-score of the signal was used for correlation analysis. Unless otherwise specified, all plots and cortical images are the average of 5 animals. For each animal, a MATLAB data structure was generated containing behavior data, filtered photometry and cortical signals, and metadata, which is openly available, along with scripts used for generating results and figures.

## 3 Results

### 3.1 Post-Surgical Welfare and Post-Mortem Histology

Combining mesoscale cortical Imaging with subcortical fiber photometry required us to position optical fibers so that they were not obscuring the cortical mesoscale imaging window. To accomplish this, we first consulted the Allen brain connectivity Atlas^15^ and determined potential insertion locations for a lateral angle approach. Previous work indicates that the striatum contains select regions that are mapped to the sensory-motor cortex.^6^ Using these anatomical landmarks, we assessed regions that are expected to receive input from the sensory cortex as described by Hunnicutt et al.,^16^ and in the Allen Brain Atlas. We also reviewed diagrams of facial musculature and other overlying structures, which could lead to potential animal care issues and worked around them. We were able to settle upon an insertion path, which was located 3 mm lateral to bregma and involved inserting the fiber at a 45 deg angle, which allowed us to reach the dorsal striatum. During pilot experiments, animals were closely monitored for any signs of pain or weight loss, which was not significantly different 7 days following the surgery from their baseline weight.

The post-mortem collection of head caps (including the screw, optical fiber, and dental cement), showed an average additional weight of 0.530±0.02  g, and the histology confirmed the diagonal insertion of the optical fiber to the dorsolateral striatum (DLS) [Figs. 3(b) and 3(c)]. It is important to note that coordinates anterior to bregma+1 mm require modifications to the cranial window preparation, as the ferrule would be close to the eye. In addition, we were able to successfully insert the optical fiber into other subcortical regions, including the thalamus and the hippocampus (unpublished data) without any complications.

The histology showed clear expression of GcaMP6s in the cortex and axon bundles in the striatum. Based on reports from the Allen Brain Atlas and the original publications that characterized the Thy1-GCAMP6s mouse line,^11^ we would expect GCAMP6 expression to include both intratelencephalic and pyramidal tract classes of pyramidal neurons. We also acknowledge that future work could extend this using more selective Cre-lines of mice to target more specific axonal projection patterns, or potentially use retrogradely transported AAVs to label neurons that project to a specific site.

To optimize the recording setup, several control experiments were done and multiple filter sets were used. The diagonal insertion of the fiber and limited dental cement surrounding the ferrule makes it fragile and less resistant to pressure and torque applied to the ferrule. Although we did not encounter any spontaneous breakage of the head cap over a 3- to 4-month period following surgery, attaching the fiber and placing the mouse under the imaging setup should be done with care. The fiber patch cord should loosely pass through multiple holding rings to minimize the movement and torque applied to the animal. The head fixing screw should be at a 25 to 30 deg angle so that there is no contact between the head restraining post and the camera lens. As a result, we highly recommend that the imaging apparatus be checked before recording using a dummy mouse or a headcap. Using a fixed-focus imaging setup keeps the scale and headcap distance constant between animals and imaging sessions. However, it requires a camera mount that can easily move vertically.

### 3.2 Control Experiments

To investigate any potential crosstalk or excitation/emission light contamination of either cortical imaging or fiber photometry recordings, we performed control recordings for green epi-fluorescence applications. In each recording, the cortical or fiber photometry excitation LED was turned on and off while continuing to monitor the signal in the other structure. For example, does cortical LED excitation light affect the emitted signal observed within the striatum (or the converse) [Figs. 3(d)–3(g)]? When the fiber LED is turned on, and the cortical overhead LED is off, we noticed ∼2% increase in the raw pixel values of the CCD camera cortical signal in a small region around the fiber insertion site [Fig. 3(a)]. This excitation light was not sufficient to produce a detectable signal, as demonstrated by low SNR [Fig. 3(d)] or the standard deviation (STD) of the raw signal in that region [Fig. 3(e)]. We also observed no significant increase in the fiber photometry signal following excitation of the cortex using the overhead LED [Figs. 3(f)–3(g)]. However, it is important that in the case of dual-color imaging the filter sets be optimized and control experiments are done to ensure absence of any crosstalk between the two channels. Furthermore, given that the excitation light for fiber photometry is modulated, based on the cortical imaging sampling rate, one should account for aliasing-dependent contamination of the cortical recordings in case of light leakage from fiber to cortex. In the case of our recordings, the aliasing frequencies (∼20 to 40 Hz) were calculated to be above the filtered output (12 Hz). In the presence of cortical excitation strobing, the same issue might result in unwanted noise in the collected fiber photometry signal that could be identified and removed by filtering. Future improvements of the method would also include applying hemodynamic corrections using optical strobing.^17^ These steps may help to reduce residual noise due to the heartbeat that may be visible at ∼10  Hz.^18^

### 3.3 Sensory-Evoked Responses in Cortex and Striatum

To validate the functional neural responses in the cortex and striatum, visual and auditory sensory responses were evoked as described in the methods section. During a 6 to 8-min recording session, 40 stimuli were provided with an ISI of 10 s. The average of the first 10 responses was calculated and compared to the average of the last 10 evoked responses to investigate any habituation to the stimuli.

**Fig. 4:**
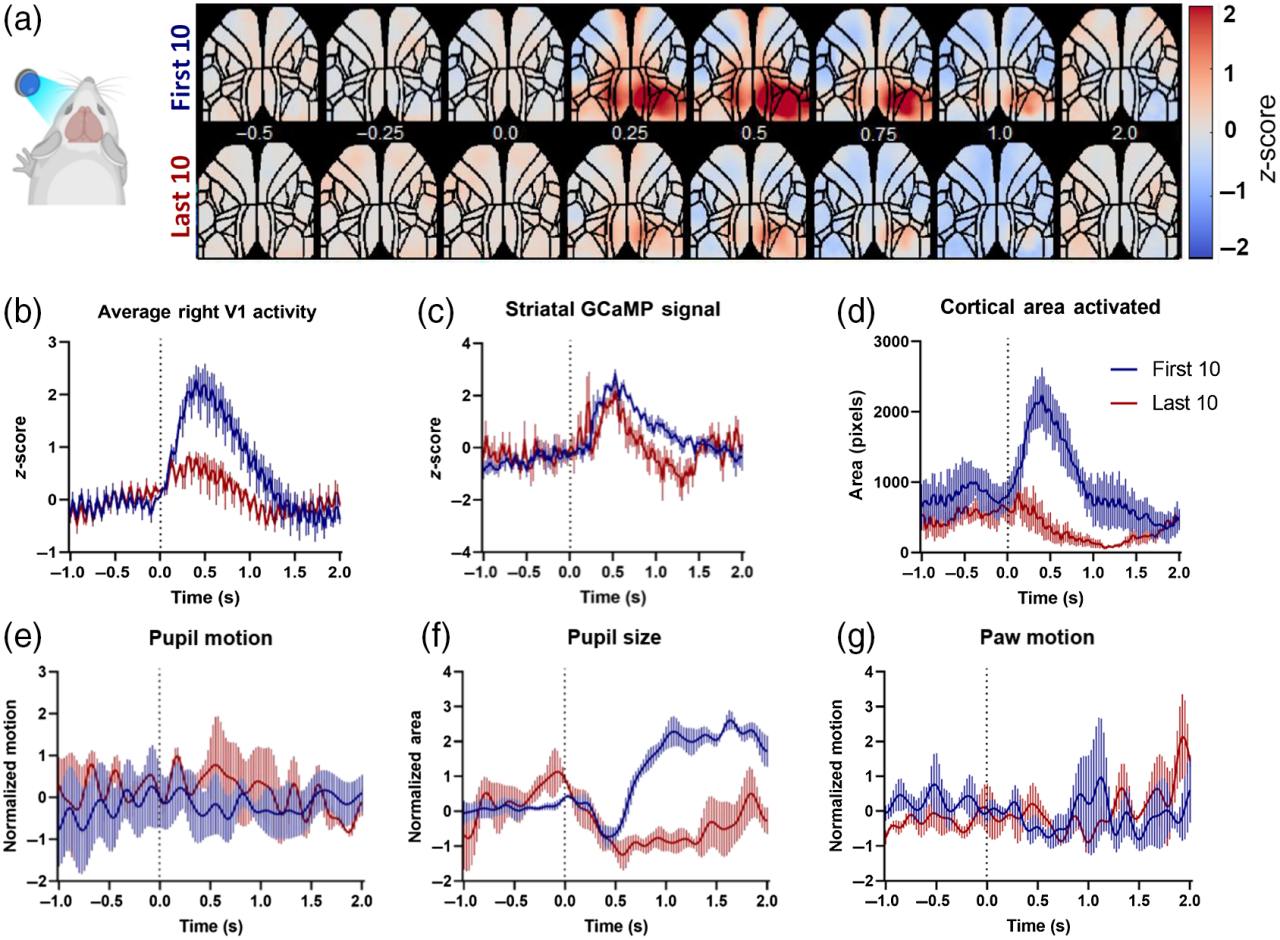
Visually evoked cortical, striatal, and motor responses in Thy-1 GCAMP6 mice. (a) Average of first 10 (top panel) and last 10 (bottom panel) cortical responses to visual stimulation (n=5 animals), showing a reduction of visually evoked responses. (b) Average activation of the right primary visual cortex in response to left visual field stimulation. (c) Average striatal presynaptic (axon terminal) activity following the contralateral visual stimulation. (d) The cortical area activated following visual stimulation, calculated as number of pixels above mean + 4*STD of baseline (1 s before stimulation). (e)–(g) Average motor responses of the pupil (size and movement) and forepaw movement, normalized to the baseline (1 s before the stimulation).

**Fig. 5:**
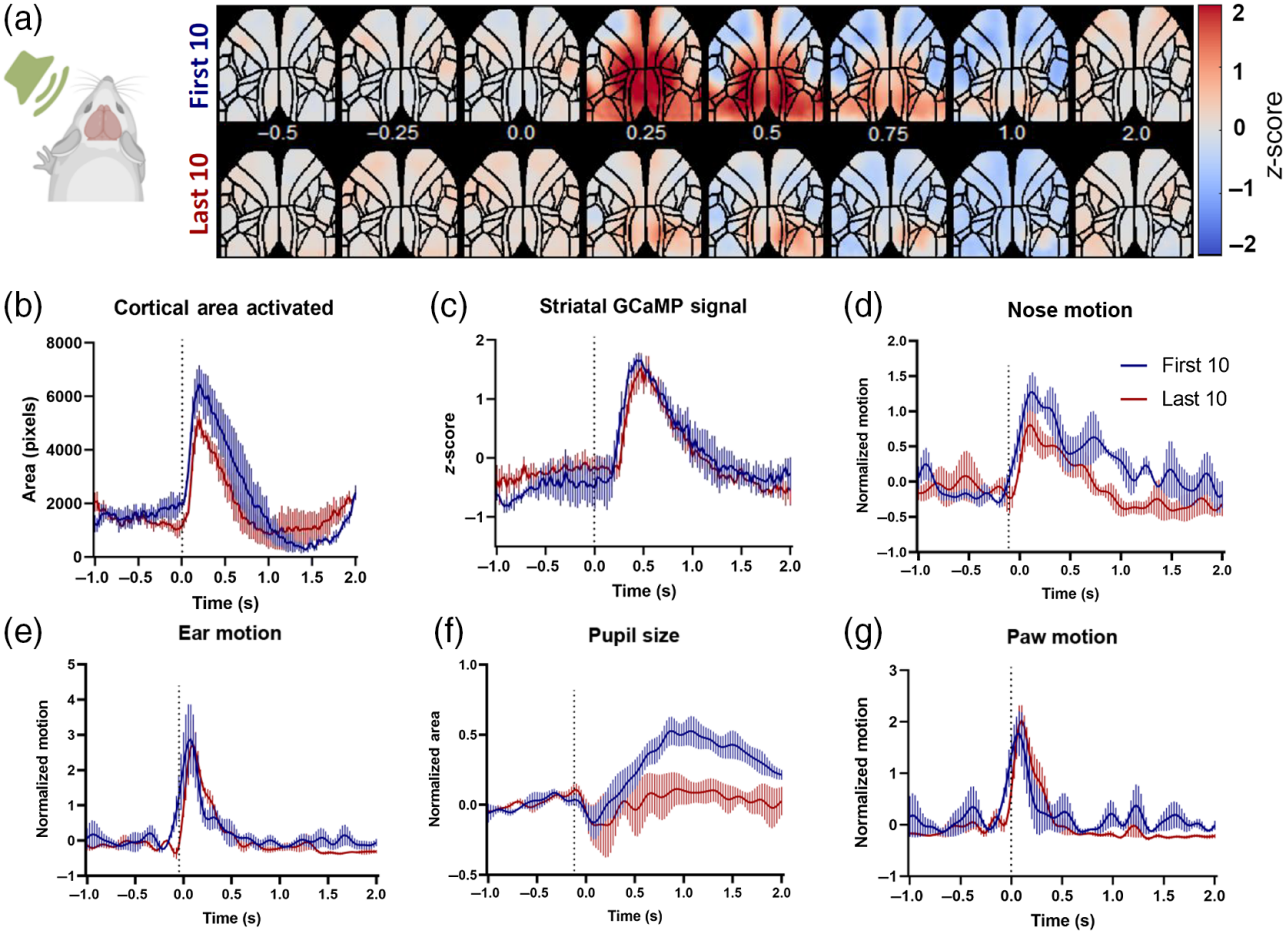
Auditory-evoked cortical, striatal, and motor responses. (a) Average of first 10 (top panel) and last 10 (bottom panel) cortical responses to auditory stimulation (n=5 animals), showing a moderate reduction of auditory-evoked responses. (b) The cortical area activated following visual stimulation, calculated as number of pixels above mean + 4*STD of baseline (1 s before stimulation). (c) Average striatal presynaptic (axon terminal) activity following the auditory stimulation. (d)–(g): Average motor responses of the nose, ear, pupil size, and forepaw movement, normalized to the baseline (1 s before the stimulation).

We noticed strong habituation to the visual stimuli (Fig. 4) and moderate habituation to the auditory stimuli (Fig. 5). The expected axon terminal calcium responses in the striatum showed a moderate decrease for the visual [Fig. 4(c)] and no change for the auditory stimulation [Fig. 5(c)]. The visual stimuli evoked well-defined unilateral responses in visual cortical regions [Fig. 4(a)], which showed a reduction in amplitude [Fig. 4(b)] and spread [Fig. 4(d)] in the last 10 stimuli. The auditory evoked cortical responses spread bilaterally throughout the cortices including the sensorimotor areas [Fig. 5(a)]. The spread of these evoked signals in the cortex decayed moderately during the imaging session [Figs. 5(a) and 5(b)]. Interestingly, the amplitude and decay of the striatal axon terminal signal did not show any changes [Fig. 5(c)].

The behavior videos showed a reduction in the pupillary responses in the last 10 stimuli (both visual and auditory: Figs. 4(f) and 5(f), suggesting sensory habituation and less arousal. No significant rapid movement in any body part was recorded following visual stimulations [Figs. 4(e) and 4(g)]. However, all animals consistently showed startle-like movements in the ears, paws, and nose following auditory stimulation [Figs. 5(d), 5(e), and 5(g)]. This reflex did not reduce across the 40-stimuli session.

### 3.4 Spontaneous Cortical GCaMP Activity

**Fig. 6:**
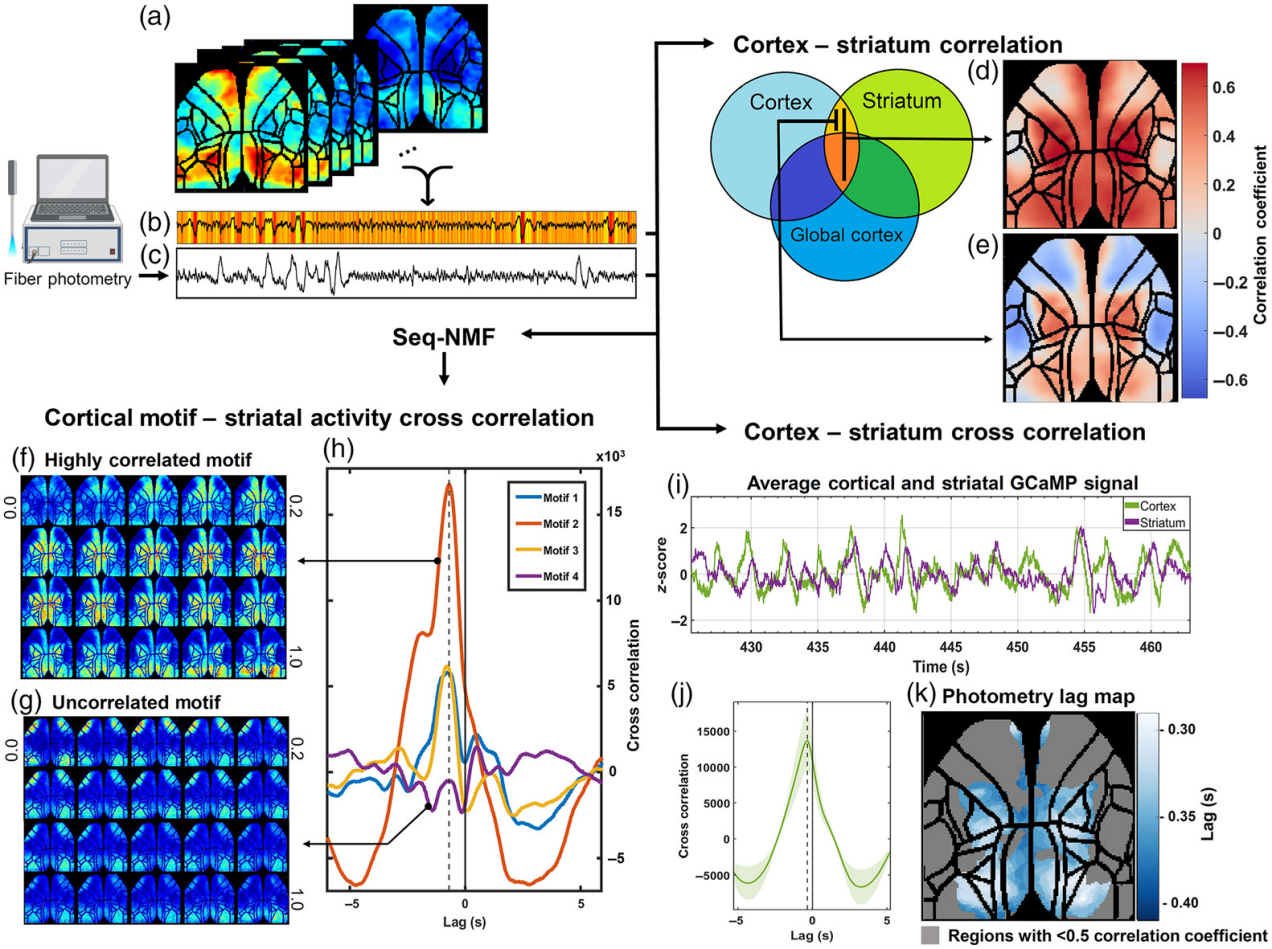
Data analysis pipeline and correlation analysis of the spontaneous corticostriatal activity. (a) Cortical images were imported as temporal stacks of X/Y frames. (b) The global cortical activity was calculated as the spatial average over all cortical pixels. The correlation coefficient of each cortical pixel with the photometry signal (c), was used to calculate full signal correlation map (d). The same process was done for each pixel after regressing out the global cortical activity to generate the partial correlation map (e). The correlation maps shown are averaged for all animals (n=5 animals). (f) and (g) Repeating patterns of cortical activity (motifs) were detected with seq-NMF^13^ (from left to right, top to bottom, total of 1 s). (f) An example motif showing anterior to posterior activation of the sensorimotor regions of the cortex, with similar pattern to the correlation maps. (g) An example motif from the same animal, which showed low correlation and consisted of activation of anterolateral motor regions (ALM), and visual cortices. (h) A representative cross-correlation of the motif loadings with the striatal signal. (i) A representative trace of the average cortical activity showing the lag between cortical activity and striatal presynaptic activity. (j) The average time lag was calculated to be ∼300  ms (cortex leads striatum; n=5 animals). (k) The time lag with maximum cross-correlation was calculated for each pixel to generate the photometry lag map (averaged for all animals; n=5).

To measure the correlation between optical signals collected from the mesoscale cortex and point-source striatum, animals were head-fixed and imaged for 20 min. The cortical images were imported as a series of frames over time [Fig. 6(a)], and the average activity across the cortical mask was then calculated as a measure of global cortical activity [Fig. 6(b)]. Correlation maps were calculated as a pixel-wise correlation (or partial correlation) of cortical with striatal activity [collected through fiber photometry: Fig. 1(c)], either without [Fig. 6(d)] or with [Fig. 6(e)] regression of the cortical global activity.^18^ Interestingly, a similar correlation pattern between dorsal striatum and cortex was observed across all animals and was consistent with cortical sensorimotor networks.

GCaMP cortical signals showed 5 to 10 distinct repeating patterns of activity (motifs) for each animal studied [Figs. 6(f) and 6(g)].^19^^,^^20^ Some of these cortical motifs had similar spatial characteristics as the “butterfly-like” pattern of cortico-striatal correlation [compare Figs. 6(f) to 6(e)], whereas others showed activated regions outside of the cortico-striatal correlation map [Fig. 6(g)]. These two major classes of motifs [Figs. 6(f) and 6(g)] were observed consistently in all animals studied. The motifs with the similar butterfly-like pattern had higher cross-correlation peaks (inner dot product), than motifs that did not contain activity in the regions observed in the overall correlation of the cortex and striatum [Fig. 6(h)]. The relative time lag in the motif loadings was ∼700  ms [Fig. 6(h)], showing that this cortical motif precedes striatal terminal activity. It is worth noting that the motif loadings are convolved with the 1-s motif [Figs. 6(f) and 6(g)], making the exact value of the delay unclear.

The global cortical activity also showed a negative time lag relative to the axon terminal activity recorded in the striatum (∼300  ms), indicating that cortical somatodendritic activity generally precedes the calcium activity in the (cortical and/or thalamic) axon terminals in the striatum [Figs. 6(i) and 6(j)]. The lag map [Fig. 6(k)] shows the time lag (average of n=5 animals) associated with the maximum cross correlation of each pixel with the presynaptic striatal signal. Regions colored gray show correlation coefficients lower than 0.5, and no detectible peak in the cross-correlation analysis. On average, posterior regions of cortex have lower time lag (∼−0.3  s) as opposed to more anterior parts of the somatosensory regions (∼−0.4  s). This is in line with the anterior to posterior flow of cortical processing observed in the discovered motifs.

## 4 Discussion

We developed and optimized a novel technique to measure brain activity at multiple levels, using optical fiber photometry and widefield cortical imaging. Using genetically encoded optical sensors is preferred in many study designs as it can provide extensive flexibility (in terms of the color spectrum and chemical components being measured) and specificity (in terms of population of cells being studied).

Our control experiments showed that cortical imaging and fiber photometry can be combined with minimal crosstalk. We have employed relatively large diameter fibers (400  μm) to test the potential upper bounds of optical crosstalk between modulated striatal fiber signals and cortical wide-field imaging. In preliminary experiments, similar signals were obtained using 200-μm fibers and would be expected to be sufficient for this method (data not shown).

We were able to validate our method using well-described sensory-evoked responses in awake mice. In awake animals, visual and auditory stimulation evoked responses in cortex and striatum. Given the different habituation of the signal in the cortex and the striatum, it is likely that striatal responses (especially to the visual stimuli) are partially derived from thalamic activity. It has previously been shown that cortical afferents from the visual cortex can be tracked to the anterior striatum and sensory-evoked responses can be recorded at single-cell or population level.^6^^,^^21^ In this study, we examined visual and auditory sensory responses, but it can be utilized to study both laminar and lateral sensory spread in the barrel cortex and its relation to subcortical thalamic inputs.^22^ Furthermore, this method is especially useful in investigating cortical circuit remapping following stroke, as it provides insight to the circuit at multiple scales including sub-cortical sites.^23^

We provide evidence that the presynaptic (axon terminal) responses in the striatum undergo different habituation compared to the cortical somatodendritic responses. Although the basis for these evoked responses has not been fully resolved, most projections to the dorsal striatum originate from sensorimotor regions of the cortex,^16^ and thus, the sensory-evoked responses in the striatum are entirely due to activation of corticostriatal inputs. However, our observations are in line with Mowery et al.,^24^ who performed simultaneous extracellular recording of DLS and primary sensory cortex in lightly anesthetized rats, showing an important thalamic component in the striatal sensory-evoked responses. Furthermore, the stronger habituation of upstream cortical than downstream striatal optical signals is interesting but needs to be taken in the context of a multi-layered cortex. Potentially, habituation within layers 1 and 2/3 where the bulk of single-photon widefield signals were measured^25^ is potentially different from that of output projections from layer 5 and 6 neurons to striatum where presynaptic axonal signals were measured. Note that most of the studies showing direct and single-origin corticostriatal sensory responses are performed on deeply anesthetized rodents, whose thalamostriatal responses are highly suppressed due to the anesthesia.^26^ Mowery et al.^24^ observed that sensory barrel cortex responses to whisker stimulation show rapid habituation to repeated stimuli presentation, whereas MSN responses in the DLS, as well as in the thalamus, habituate very slowly to the stimuli. In the other studies providing similar findings, a multiscale or multilevel approach has been utilized, underlining the importance of such techniques. Using these multiscale methods in neuroimaging will provide a richer and more thorough view of the brain activities underlying behavior. Combined with our head-fixed social interaction apparatus,^27^ this method may provide richer insights into cortical and hypothalamic networks in social interaction.^28^^,^^29^

To study the correlation between corticostriatal recording and behavior, we measured brain activity and behavior in head-fixed mice. We showed that average cortical activity and striatal terminal activity are highly correlated. Interestingly, a lag between cortical and striatal activity was observed, which was in line with the study of Peters et. al.^6^ Although our data indicates that cortical somatodendritic activity preceeds terminal activation in the striatum during spontaneous activity, it should be noted that this cross-correlation time lag is heavily dependent on the method of recording (optical versus electrical and the kinetics of the optical sensor), and therefore should not be used to infer exact delays in corticostriatal signal transmission.

The cortical networks interact locally and globally across scales. Recent studies have begun to document such multiscale interactions. One main goal of combining fiber photometry and widefield cortical imaging is to better investigate the basal outputs of local cortical activity. We utilized an unsupervised method (seq-NMF^13^) to discover spatiotemporal motifs of cortical activity and showed that specific motifs are correlated with excitatory terminal activation in the striatum. The motifs recovered with seq-NMF were very similar to those previously reported by others and shown to be linked to sensory stimuli or other behaviorally relevant events.^12^^,^^20^ This combination provides an insight into the interactions between cortical processing of sensorimotor stimuli, and activation of basal ganglia. Given the heterogeneity of the signal we recorded from the striatum (being cortical or thalamic inputs), it is not possible to distinguish between the networks.

Cortical imaging of fluorescent genetic probes for neurotransmitters or cell-type-specific expression of GECIs will allow for more detailed tracking of multiscale cortical processing of information. Recently, multiple studies have exploited probes to measure neurotransmitters/modulators in spontaneous brain activity.^30^^–^^32^ Combining this method with single-cell microprobes^33^ would also enable parallel single-cell optoelectric recordings in the context of mesoscale cortical wide-field imaging. Understanding the relationship between brain activity across local and global networks requires multiscale recording approaches that are compatible with genetically encoded optical sensors such as mesofiber dual cortical and striatal assessment. These approaches may help clarify the neural correlates of behavior, and mechanisms underlying learning with specific cell populations and projection targets.

## Code, Data, and Materials Availability

All the codes used for data analysis and sample wide-field calcium imaging data generated in this study have been deposited in the Open Science Framework (OSF) repository (https://osf.io/jc5t9/).
